# Supplementary material for: A new phylogenetic data standard for computable clade definitions: the Phyloreference Exchange Format (Phyx)
Source: PeerJ. 2022 Feb 15;10:e12618. doi: 10.7717/peerj.12618 (PMC8855714; doi:10.7717/peerj.12618)
Supplement: Supplemental Information 1 — Our definition of a Citation object is based on the BibJSON format (http://okfnlabs.org/bibjson/), which is itself based on the BibTeX format (Patashnik, 1988). All of the fields in the table come from those formats, except for “bibliographicCitation”, which is defined in the Dublin Core standard (http://purl.org/dc/terms/bibliographicCitation). Note that only the “bibliographicCitation” field will carry over to RDF (and thus OWL ontology) transformation. If it does not have a value, the phyx.js library will compute its value from the other fields. [file peerj-10-12618-s001.docx]

**Supplemental Table S1: Fields in each Citation object.** Our definition of a Citation object is based on the BibJSON format (<http://okfnlabs.org/bibjson/>), which is itself based on the BibTeX format [(Patashnik, 1988)](https://paperpile.com/c/Yi0Orh/zTuF). All of the fields in the table come from those formats, except for “bibliographicCitation”, which is defined in the Dublin Core standard (<http://purl.org/dc/terms/bibliographicCitation>). Note that only the “bibliographicCitation” field will carry over to RDF (and thus OWL ontology) transformation. If it does not have a value, the phyx.js library will compute its value from the other fields.

| **Field name** | **Description** | **Type** | **Example** |
| --- | --- | --- | --- |
| bibliographicCitation | A bibliographic reference for the resource. If not included, one will be generated from the other fields in this citation. | String | Kirsten M. Fisher, Dennis P. Wall, Kwok Leung Yip, Brent D. Mishler (1 March 2007) Phylogeny of the Calymperaceae with a rank-free systematic treatment. The Bryologist, 110(1):46-73, doi: 10.1639/0007-2745(2007)110[46:POTCWA]2.0.CO;2 |
| type | The type of this citation. Should be one of the BibTeX entry types (https://www.bibtex.com/e/entry-types/), such as “article”, “book”, “inbook” and so on. | String | article |
| title | The title of this publication. | String | Integrating molecular phylogenetic and paleobotanical evidence on origin of the flower. |
| year | The year of publication. | Integer | 2008 |
| authors | A list of authors of this publication. Each author is described with the following fields: | Array of Objects |  |
| - name | The full name of the author. | String | J. A. Doyle |
| - alternate | A list of alternate representations of the author name. | Array of Strings | [ “Doyle, J. A.” ] |
| - firstname | The first (or given) name of the author. | String | J. |
| - middlename | The middle name of the author. | String | A. |
| - lastname | The last (or family) name of the author. | String | Doyle |
| editors | A list of editors, in the same format as the authors. | Array of Objects | See author example above. |
| series_editors | A list of series editors, in the same format as the authors. | Array of Objects | See author example above. |
| identifier | A list of identifiers, each of which has the following properties: | Array of Objects |  |
| - type | **Required.** The type of identifier. | String | doi, issn |
| - id | **Required.** The identifier itself. | String | 10.1086/589887, 0002-9327 |
| link | A list of links with the following properties: | Array of Objects |  |
| - url | **Required.** A URL to this article | URL | <https://www.journals.uchicago.edu/doi/10.1086/589887> |
| journal | An object containing information on the journal this publication belongs to. A journal can have the following properties: | Object |  |
| - name | **Required.** The journal name. | String | International Journal of Plant Science |
| - volume | The journal volume. | String | 169 |
| - number | The issue number. | String | 7 |
| - pages | The page numbers of the journal that this citation refers to. | String | 816--843 |
| - identifier | A list of identifiers (for the journal), in the same format as the “identifier” properties above. | Array of Objects | [ {    "id": "0002-9327", "type": "issn"  } ] |
| booktitle | The title of the book that this publication is part of. | String | Phylonyms |
| publisher | The publisher of this publication. | String | The University of Chicago Press |
| city | The city in which this publication was published. | String | Chicago, IL |
| pages | The page numbers of this publication that this citation should refer to. | String | 816--843 |
| figure | The figures this citation refers to. | String | 3C, 3A, 4 |
