# Supplementary material for: A new phylogenetic data standard for computable clade definitions: the Phyloreference Exchange Format (Phyx)
Source: PeerJ. 2022 Feb 15;10:e12618. doi: 10.7717/peerj.12618 (PMC8855714; doi:10.7717/peerj.12618)
Supplement: Supplemental Information 2 — All of these fields are from the “Occurrence” section of the Darwin Core standard (https://dwc.tdwg.org/terms/#occurrence), except for “hasName”, which is taken from the TDWG Taxon Concept LSID Ontology (http://rs.tdwg.org/ontology/voc/TaxonConcept), part of the TDWG Ontology (https://github.com/tdwg/ontology). [file peerj-10-12618-s002.docx]

**Supplemental Table S2: Fields in a specimen object.** All of these fields are from the “Occurrence” section of the Darwin Core standard (<https://dwc.tdwg.org/terms/#occurrence>), except for “hasName”, which is taken from the TDWG Taxon Concept LSID Ontology ([http://rs.tdwg.org/ontology/voc/TaxonConcept](https://github.com/tdwg/ontology/blob/master/ontology/voc/TaxonConcept.rdf)), part of the TDWG Ontology (<https://github.com/tdwg/ontology>).

| **Field name** | **Description** | **Type** | **Example** |
| --- | --- | --- | --- |
| @type | **Required.** Our terminology for specimens comes from the Darwin Core standard [(Wieczorek et al., 2012)](https://paperpile.com/c/Yi0Orh/bqWl). | IRI | Must be <http://rs.tdwg.org/dwc/terms/Occurrence> |
| @id | An identifier for this specimen. | IRI | <https://www.wikidata.org/wiki/Q1514294> |
| basisOfRecord | The type of specimen indicated. | String | PreservedSpecimen |
| institutionCode | The name (or acronym) in use by the institution having custody of the object(s) or information referred to in the record. | String | FMNH |
| collectionCode | The name, acronym, code, or initialism identifying the collection or data set from which the record was derived. | String | PR |
| catalogNumber | An identifier for the record within the data set or collection. | String | 2081 |
| occurrenceID | An identifier for the Occurrence (as opposed to a particular digital record of the occurrence). In the absence of a persistent GUID, constructed from a unique combination of identifiers in the record, usually the Darwin Core Triplets (<https://dwc.tdwg.org/rdf/>) of institutionCode, collectionCode and catalogNumber. | String | FMNH:PR:2081 |
| hasName | Can be used to record the taxon name that this specimen has been identified to. | Taxon Name | See example of a Taxon Name in the *Taxon or taxon concept* section in the main text. |
