# Supplementary material for: A new phylogenetic data standard for computable clade definitions: the Phyloreference Exchange Format (Phyx)
Source: PeerJ. 2022 Feb 15;10:e12618. doi: 10.7717/peerj.12618 (PMC8855714; doi:10.7717/peerj.12618)
Supplement: Supplemental Information 4 [file peerj-10-12618-s004.pdf]

Introduction to phyx.js

Written by Gaurav Vaidya. Last updated August 24, 2021

This tutorial provides an introduction to the phyx.js library, and shows you how it can be used to read a Phyx file, check it for validity, examine phylorefences, phylogenies and specifiers, and describe how to convert the file into RDF.

Starting with Phyx files

Phyx files are digitized clade definitions in a JSON-LD format. While you can use an editor like Klados to create Phyx files, you can also write one by yourself as a JSON document.

```
var alligatoridae_brochu2003 = {
    "@context": "https://www.phyloref.org/phyx.js/context/v1.0.0/phyx.json",

    // Phylogeny from Brochu 2003: https://doi.org/10.1146/annurev.earth.31.100901.141308
    "phylogenies": [{
        "newick": "(Parasuchia,(rauisuchians,Aetosauria,(sphenosuchians,(protosuchians,(mesosuchians,(Hylaeochampsa,Aegyptosuchus,Stomatosuchus,(Allodaposuchus,('Gavialis gangeticus',(('Diplocynodon ratelii',('Alligator mississippiensis','Caiman crocodilus')Alligatoridae)Alligatoroidea,('Tomistoma schlegelii',('Osteolaemus tetraspis','Crocodylus niloticus')Crocodylinae)Crocodylidae)Brevirostres)Crocodylia))Eusuchia)Mesoeucrocodylia)Crocodyliformes)Crocodylomorpha));",
        "source": {
            "type": "article",
            "title": "Phylogenetic approaches toward crocodylian history",
            "authors": [{
                "firstname": "Christopher",
                "middlename": "A.",
                "lastname": "Brochu"
            }],
            "year": 2003,
            "figure": "1",
            "identifier": [{
                "type": "doi",
                "id": "10.1146/annurev.earth.31.100901.141308"
            }],
            "journal": {
                "name": "Annual Review of Earth and Planetary Sciences",
                "volume": "31",
                "pages": "357--397",
                "identifier": [{ "type": "eISSN", "id": "1545-4495" }]
            }
        }
    }],

    // Clade definition from Brochu 2003: Alligatoridae
```

```
    "phylorefs": [{
        "label": "Alligatoridae",
        "scientificNameAuthorship": { "bibliographicCitation": "(Cuvier 1807)" },
        "phylorefType": "phyloref:PhyloreferenceUsingMinimumClade",
        "definition": "Alligatoridae (Cuvier 1807).\n\nLast common ancestor of Alligator mississippiensis and Caiman crocodilus and all of its descendents.",
        "definitionSource": {
            "bibliographicCitation": "Brochu (2003) Phylogenetic approaches toward crocodylian history. Annual Review of Earth and Planetary Sciences 31:1, 357-397. doi: 10.1146/annurev.earth.31.100901.141308"
        },
        "internalSpecifiers": [
            {
                "@type": "http://rs.tdwg.org/ontology/voc/TaxonConcept#TaxonConcept",
                "hasName": {
                    "@type": "http://rs.tdwg.org/ontology/voc/TaxonName#TaxonName",
                    "nomenclaturalCode": "http://rs.tdwg.org/ontology/voc/TaxonName#ICZN",
                    "label": "Caiman crocodilus Linnaeus, 1758",
                    "nameComplete": "Caiman crocodilus",
                    "genusPart": "Caiman",
                    "specificEpithet": "crocodilus"
                }
            }, {
                "@type": "http://rs.tdwg.org/ontology/voc/TaxonConcept#TaxonConcept",
                "hasName": {
                    "@type": "http://rs.tdwg.org/ontology/voc/TaxonName#TaxonName",
                    "nomenclaturalCode": "http://rs.tdwg.org/ontology/voc/TaxonName#ICZN",
                    "label": "Alligator mississippiensis (Daudin, 1802)",
                    "nameComplete": "Alligator mississippiensis",
                    "genusPart": "Alligator",
                    "specificEpithet": "mississippiensis"
                }
            }
        ]
    }]
}
```

Validating a Phyx document using JSON Schema

We publish a JSON Schema with phyx.js, which can be used to validate that a Phyx document is correctly formed. We use Ajv, a JSON Schema validator for JavaScript. Note that we use the copy of the context file that is included in this repository, but you can also download it from our website.

```
var fs = require('fs');
var Ajv = require('ajv');

// Configure Ajv.
var ajv = new Ajv({
    allErrors: true, // Display all error messages, not just the first.
});

// We use the JSON Schema included with this repository, but you can download the
// Phyx JSON Schema from https://www.phyloref.org/phyx.js/context/v1.0.0/schema.json
var validator = ajv.compile(JSON.parse(fs.readFileSync('../docs/context/v1.0.0/schema.json')));

// Attempt to validate the Brochu 2003 example file.
var result = validator(alligatoridae_brochu2003);
console.log(`Is alligatoridae_brochu2003 valid? ${result}`);
console.log('Errors:', validator.errors || 'none');

// Let's make an invalid copy of the Brochu 2003 example file to make sure this is working.
var alligatoridae_brochu2003copy = {...alligatoridae_brochu2003};
delete alligatoridae_brochu2003copy['@context'];  // @context is required for successful validation

var result = validator(alligatoridae_brochu2003copy);
console.log(`Is alligatoridae_brochu2003copy valid? ${result}`);
console.log('Errors:', validator.errors);
```

```
Is alligatoridae_brochu2003 valid? true
Errors: none
Is alligatoridae_brochu2003copy valid? false
Errors: [
  {
    keyword: 'required',
    dataPath: '',
    schemaPath: '#/required',
    params: { missingProperty: '@context' },
    message: "should have required property '@context'"
  }
]
```

Examining phylorefs, taxonomic units and taxon concepts

The phyx.js library was built in order to simplify the process of working with individual components of Phyx documents, and to facilitate the conversion of a Phyx document into OWL. The library consists of a series of wrappers, each of which wraps part of the document. For example, we can wrap every specifier that is a taxonomic unit using the TaxonomicUnitWrapper.

This provides a number of convenience methods: for example, `.internalSpecifiers` and .

`externalSpecifiers` will always return lists, whether or not these are defined in the underlying phyloreference (if undefined, the methods return empty lists). There is also a `.specifiers` method that lists both internal and external specifiers.

Furthermore, taxonomic units that are taxon concepts can be wrapped by a TaxonConceptWrapper, which have methods for accessing the “complete name” (i.e. the monomial, binomial or trinomial name) and the nomenclatural code.

```
// Load the Phyx library.
var phyx = require('..');

// List all the phylorefences along with their specifiers.
alligatoridae_brochu2003.phylorefs.forEach(phyloref => {
    let wrappedPhyloref = new phyx.PhylorefWrapper(phyloref);

    console.log(wrappedPhyloref.label);

    // Extract the "complete name" and the nomenclatural code short name for each specifier that is a taxonomic unit.
    wrappedPhyloref.internalSpecifiers.forEach(specifier => {
        let wrappedSpecifier = new phyx.TaxonomicUnitWrapper(specifier);
        if (wrappedSpecifier.taxonConcept) {
            let wrappedTaxonConcept = new phyx.TaxonConceptWrapper(wrappedSpecifier.taxonConcept);
            console.log(` - Internal: ${wrappedTaxonConcept.nameComplete} (${wrappedTaxonConcept.nomenCodeDetails.shortName})`);
        } else {
            console.log(` - Internal: ${wrappedSpecifier.label}`);
        }
    });

    // Note that the phyloref doesn't have an `externalSpecifiers` key, but the wrapper provides it as an empty list
    // for ease of use.
    wrappedPhyloref.externalSpecifiers.forEach(specifier => {
        let wrappedSpecifier = new phyx.TaxonomicUnitWrapper(specifier);
        if (wrappedSpecifier.taxonConcept) {
            let wrappedTaxonConcept = new phyx.TaxonConceptWrapper(wrappedSpecifier.taxonConcept);
            console.log(` - External: ${wrappedTaxonConcept.nameComplete} (${wrappedTaxonConcept.nomenCodeDetails.shortName})`);
        } else {
            console.log(` - External: ${wrappedSpecifier.label}`);
        }
    });

    console.log();
});
```

```
Alligatoridae
 - Internal: Caiman crocodilus (ICZN)
 - Internal: Alligator mississippiensis (ICZN)
```

Examining phylogenies

Phylogenies are stored in JSON files as Newick strings, but the PhylogenyWrapper can be used to look at internal and terminal node labels and to translate the Newick string into a JSON structure for use by downstream programs.

```
var phylogeny = alligatoridae_brochu2003.phylogenies[0];
console.log(`The phylogeny is represented by the Newick string: ${phylogeny.newick}`);
console.log();

// Display internal and external nodes.
var wrappedPhylogeny = new phyx.PhylogenyWrapper(phylogeny);
console.log(`This consists of the following nodes:\n - Internal nodes: ${wrappedPhylogeny.getNodeLabels('internal').join(', ')}`);
console.log(` - Terminal nodes: ${wrappedPhylogeny.getNodeLabels('terminal').join(', ')}`);
console.log();

// Convert the Newick string into a JSON structure for examination.
console.log(`Newick string as a JSON structure: ${JSON.stringify(phyx.PhylogenyWrapper.getParsedNewick(phylogeny.newick), undefined, 2)}`);
console.log();
```

```
The phylogeny is represented by the Newick string: (Parasuchia,(rauisuchians,Aetosauria,(sphenosuchians,(protosuchians,(mesosuchians,(Hylaeochampsa,Aegyptosuchus,Stomatosuchus,(Allodaposuchus,('Gavialis gangeticus',(('Diplocynodon ratelii',('Alligator mississippiensis','Caiman crocodilus')Alligatoridae)Alligatoroidea,('Tomistoma schlegelii',('Osteolaemus tetraspis','Crocodylus niloticus')Crocodylinae)Crocodylidae)Brevirostres)Crocodylia))Eusuchia)Mesoeucrocodylia)Crocodyliformes)Crocodylomorpha));

This consists of the following nodes:
 - Internal nodes: Alligatoridae, Alligatoroidea, Crocodylinae, Crocodylidae, Brevirostres, Crocodylia, Eusuchia, Mesoeucrocodylia, Crocodyliformes, Crocodylomorpha
 - Terminal nodes: Parasuchia, rauisuchians, Aetosauria, sphenosuchians, protosuchians, mesosuchians, Hylaeochampsa, Aegyptosuchus, Stomatosuchus, Allodaposuchus, Gavialis gangeticus, Diplocynodon ratelii, Alligator mississippiensis, Caiman crocodilus, Tomistoma schlegelii, Osteolaemus tetraspis, Crocodylus niloticus

Newick string as a JSON structure: {
  "json": {
    "children": [
      {
        "children": [
          {
            "label": "Crocodylomorpha",
            "children": [
              {
                "label": "Crocodyliformes",
                "children": [
                  {
```

```
                    "label": "Mesoeucrocodylia",
                    "children": [
                      {
                        "label": "Eusuchia",
                        "children": [
                          {
                            "children": [
                              {
                                "label": "Crocodylia",
                                "children": [
                                  {
                                    "label": "Brevirostres",
                                    "children": [
                                      {
                                        "label": "Crocodylidae",
                                        "children": [
                                          {
                                            "label": "Crocodylinae",
                                            "children": [
                                              {
                                                "label": "Crocodylus niloticus",
                                                "name": "Crocodylus niloticus"
                                              },
                                              {
                                                "label": "Osteolaemus tetraspis",
                                                "name": "Osteolaemus tetraspis"
                                              }
                                            ],
                                            "name": "Crocodylinae"
                                          },
                                          {
                                            "label": "Tomistoma schlegelii",
                                            "name": "Tomistoma schlegelii"
                                          }
                                        ],
                                        "name": "Crocodylidae"
                                      },
                                      {
                                        "label": "Alligatoroidea",
                                        "children": [
                                          {
                                            "label": "Alligatoridae",
                                            "children": [
                                              {
                                                "label": "Caiman crocodilus",
                                                "name": "Caiman crocodilus"
                                              },
                                              {
                                                "label": "Alligator mississippiensis",
                                                "name": "Alligator mississippiensis"
                                              }
                                            ],
```

```
                                              "name": "Alligatoridae"
                                            },
                                            {
                                              "label": "Diplocynodon ratelii",
                                              "name": "Diplocynodon ratelii"
                                            }
                                          ],
                                          "name": "Alligatoroidea"
                                        }
                                      ],
                                      "name": "Brevirostres"
                                    },
                                    {
                                      "label": "Gavialis gangeticus",
                                      "name": "Gavialis gangeticus"
                                    }
                                  ],
                                  "name": "Crocodylia"
                                },
                                {
                                  "label": "Allodaposuchus",
                                  "name": "Allodaposuchus"
                                }
                              ]
                            },
                            {
                              "label": "Stomatosuchus",
                              "name": "Stomatosuchus"
                            },
                            {
                              "label": "Aegyptosuchus",
                              "name": "Aegyptosuchus"
                            },
                            {
                              "label": "Hylaeochampsa",
                              "name": "Hylaeochampsa"
                            }
                          ],
                          "name": "Eusuchia"
                        },
                        {
                          "label": "mesosuchians",
                          "name": "mesosuchians"
                        }
                      ],
                      "name": "Mesoeucrocodylia"
                    },
                    {
                      "label": "protosuchians",
                      "name": "protosuchians"
                    }
                  ],
                  "name": "Crocodyliformes"
                },
                {
                  "label": "sphenosuchians",
```

```
                "name": "sphenosuchians"
              }
            ],
            "name": "Crocodylomorpha"
          },
          {
            "label": "Aetosauria",
            "name": "Aetosauria"
          },
          {
            "label": "rauisuchians",
            "name": "rauisuchians"
          }
        ]
      },
      {
        "label": "Parasuchia",
        "name": "Parasuchia"
      }
    ]
  }
}
```


Accessing citations

Another example of a wrapper that can be used for wrapping a part of a Phyx file is the CitationWrapper. This can be used to wrap citations anywhere in the Phyx file to provide a full bibliographic citation for the citation.

```
var wrappedPhylogenyCitation = new phyx.CitationWrapper(alligatoridae_brochu2003.phylogenies[0].source);
console.log(`The source of the phylogeny in this Phyx document is: ${wrappedPhylogenyCitation.toString()}`);

var wrappedPhylorefCitation = new phyx.CitationWrapper(alligatoridae_brochu2003.phylorefs[0].definitionSource);
console.log(`The definition source of the phyloreference in this Phyx document is: ${wrappedPhylorefCitation.toString()}`);
```

```
The source of the phylogeny in this Phyx document is: Christopher A. Brochu (2003) Phylogenetic approaches toward crocodylian history Annual Review of Earth and Planetary Sciences 31:357--397  fig 1 doi: 10.1146/annurev.earth.31.100901.141308
The definition source of the phyloreference in this Phyx document is: Brochu (2003) Phylogenetic approaches toward crocodylian history. Annual Review of Earth and Planetary Sciences 31:1, 357-397. doi: 10.1146/annurev.earth.31.100901.141308
```

Converting a Phyx document into OWL

A Phyx document (which is in JSON-LD format) can be converted into OWL/RDF in the form of N-Quads by using the `PhyxWrapper` to wrap the entire Phyx document. A base URL can be specified.

```
nQuads = new phyx.PhyxWrapper(alligatoridae_brochu2003).toRDF('http://example.org/phyx-tutorial#');
nQuads.then(nq => console.log(nq.slice(0, 2500) + '...'));
```

```
<http://example.org/phyx-tutorial#phylogeny0> <http://ontology.phyloref.org/phyloref.owl#newick_expression> "(Parasuchia,(rauisuchians,Aetosauria,(sphenosuchians,(protosuchians,(mesosuchians,(Hylaeochampsa,Aegyptosuchus,Stomatosuchus,(Allodaposuchus,('Gavialis gangeticus',(('Diplocynodon ratelii','Alligator mississippiensis','Caiman crocodilus')Alligatoridae)Alligatoroidea,('Tomistoma schlegelii',('Osteolaemus tetraspis','Crocodylus niloticus')Crocodylinae)Crocodylidae)Brevirostres)Crocodylia))Eusuchia)Mesoeucrocodylia)Crocodyliformes)Crocodylomorpha));" .
<http://example.org/phyx-tutorial#phylogeny0> <http://purl.obolibrary.org/obo/CDAO_0000148> <http://example.org/phyx-tutorial#phylogeny0_node0> .
<http://example.org/phyx-tutorial#phylogeny0> <http://purl.org/dc/terms/source> _:b165 .
<http://example.org/phyx-tutorial#phylogeny0> <http://www.w3.org/1999/02/22-rdf-syntax-ns#type> <http://ontology.phyloref.org/phyloref.owl#ReferencePhylogenyEvidence> .
<http://example.org/phyx-tutorial#phylogeny0> <http://www.w3.org/2000/01/rdf-schema#isDefinedBy> _:b0 .
<http://example.org/phyx-tutorial#phylogeny0_node0> <http://purl.obolibrary.org/obo/CDAO_0000149> <http://example.org/phyx-tutorial#phylogeny0_node1> .
<http://example.org/phyx-tutorial#phylogeny0_node0> <http://purl.obolibrary.org/obo/CDAO_0000149> <http://example.org/phyx-tutorial#phylogeny0_node29> .
<http://example.org/phyx-tutorial#phylogeny0_node0> <http://purl.obolibrary.org/obo/CDAO_0000200> <http://example.org/phyx-tutorial#phylogeny0> .
<http://example.org/phyx-tutorial#phylogeny0_node0> <http://www.w3.org/1999/02/22-rdf-syntax-ns#type> <http://purl.obolibrary.org/obo/CDAO_0000140> .
<http://example.org/phyx-tutorial#phylogeny0_node10> <http://ontology.phyloref.org/phyloref.owl#has_Sibling> <http://example.org/phyx-tutorial#phylogeny0_node13> .
<http://example.org/phyx-tutorial#phylogeny0_node10> <http://purl.obolibrary.org/obo/CDAO_0000149> <http://example.org/phyx-tutorial#phylogeny0_node11> .
<http://example.org/phyx-tutorial#phylogeny0_node10> <http://purl.obolibrary.org/obo/CDAO_0000149> <http://example.org/phyx-tutorial#phylogeny0_node12> .
<http://example.org/phyx-tutorial#phylogeny0_node10> <http://purl.obolibrary.org/obo/CDAO_0000179> <http://example.org/phyx-tutorial#phylogeny0_node9> .
<http://example.org/phyx-tutorial#phylogeny0_node10> <http://purl.obolibrary.org/obo/CDAO_0000187> _:b50 .
<http://example.org/phyx-tutorial#phylogeny0_node10> <http://purl.obolibrary.org/obo/CDAO_00002...
```

```
// We can convert these N-Quads into RDF/Turtle for easier interpretation.
// We only convert a few N-Quads as a demonstration.
var N3 = require('n3');
var { Readable } = require("stream")

```

```
nQuads.then(nq => {
    someNqs = nq.slice(0, 1071)

    var streamParser = new N3.StreamParser(),
    inputStream = Readable.from([someNqs]),
    streamWriter = new N3.StreamWriter();

    inputStream.pipe(streamParser);
    streamParser.pipe(streamWriter);
    streamWriter.pipe(process.stdout);
});

undefined;
```

Navigating a Phyx document as a JSON file

Most phyx.js wrappers have been designed to help interpret the more complex parts of a Phyx file, such as the phyloreferences, specifiers, phylogenies, citations and the entire Phyx document. However, since every Phyx document is also a JSON document, much of the information in the Phyx document can be accessed sufficiently easily using standard JSON libraries. In some cases, as in the demonstration below, this requires more complex code than would be necessary by using the phyx.js wrappers.

```
// List all the phylorefs in a Phyx document.
alligatoridae_brochu2003copy.phylorefs.forEach((phyloref, index) => {
    console.log(`- Phyloref ${index + 1}. ${phyloref.label}:`);
    (phyloref.internalSpecifiers || []).forEach(specifier => {
        console.log(`  - Internal specifier: ${(specifier.hasName || {}).nameComplete}`);
    });
    (phyloref.externalSpecifiers || []).forEach(specifier => {
        console.log(`  - External specifier: ${(specifier.hasName || {}).nameComplete}`);
    });
    console.log();
});
```

```
<http://example.org/phyx-tutorial#phylogeny0> <http://ontology.phyloref.org/phyloref.owl#newick_expression> "(Parasuchia,(rauisuchians,Aetosauria,(sphenosuchians,(protosuchians,(mesosuchians,(Hylaeochampsa,Aegyptosuchus,Stomatosuchus,(Allodaposuchus,('Gavialis gangeticus',(('Diplocynodon ratelii',('Alligator mississippiensis','Caiman crocodilus')Alligatoridae)Alligatoroidea,('Tomistoma schlegelii',('Osteolaemus tetraspis','Crocodylus niloticus')Crocodylinae)Crocodylidae)Brevirostres)Crocodylia))Eusuchia)Mesoeucrocodylia)Crocodyliformes)Crocodylomorpha));";
    <http://purl.obolibrary.org/obo/CDAO_0000148> <http://example.org/phyx-tutorial#phylogeny0_node0>;
    <http://purl.org/dc/terms/source> _:b0_b165;
    a <http://ontology.phyloref.org/phyloref.owl#ReferencePhylogenyEvidence>;
    <http://www.w3.org/2000/01/rdf-schema#isDefinedBy> _:b0_b0.
- Phyloref 1. Alligatoridae:
  - Internal specifier: Caiman crocodilus
```

```
  - Internal specifier: Alligator mississippiensis
```

Looking up phyloreferences on the Open Tree of Life

An included script, `resolve.js`, can be used to resolve a phyloreference on the Open Tree of Life. This can be executed from the command line by running:

```
$ npm run resolve test/examples/correct/brochu_2003.json
```

However, this script can also be invoked from within Node.js.

```
var child_process = require('child_process');

child = child_process.spawnSync('../bin/resolve.js', ['../test/examples/correct/brochu_2003.json'], {
  encoding: 'utf-8',
  stdio: 'pipe',
});
results = JSON.parse(child.output.join('\n'));

Object.keys(results).forEach(key => {
    console.log(`- ${key}:`);
    values = results[key];
    values.forEach(value => {
        if('status' in value) {
            resolved = value['resolved'];
            console.log(`  - Resolved ${value['cladeType']} phylorefence`);
            console.log(`    to: ${resolved['@id']} (label: ${resolved['label']})`);
        } else if ('error' in value) {
            console.log(`  - Could not resolve: ${value['error']}`);
        } else {
            console.log(`  - Unable to interpret: ${JSON.stringify(value, undefined, 2)}`);
        }
    })
});
```

```
- Alligatoridae:
  - Resolved minimum phylorefence
    to: https://tree.opentreeoflife.org/opentree/argus/opentree13.4@ott195670 (label: Alligatoridae)
- Alligatorinae:
  - Resolved maximum phylorefence
    to: https://tree.opentreeoflife.org/opentree/argus/opentree13.4@ott151255 (label: Alligatorinae)
- Caimaninae:
  - Resolved maximum phylorefence
    to: https://tree.opentreeoflife.org/opentree/argus/opentree13.4@ott195671 (label: Caimaninae)
- Crocodyloidea:
  - Resolved maximum phylorefence
```

```
    to: https://tree.opentreeoflife.org/opentree/argus/opentree13.4@ott335582 (
        label: Crocodylidae)
- Crocodylidae:
  - Resolved minimum phylorefence
    to: https://tree.opentreeoflife.org/opentree/argus/opentree13.4@ott1092501
        (label: Longirostres)
- Diplocynodontinae:
  - Could not resolve: no_mrca_found:400
```

About this notebook

This document was created as a Jupyter Notebook, and the source file is available in our GitHub repository. We recommend installing Jupyterlab via Homebrew on Mac, but other installation options are available. Once Jupyter Notebook is set up, you should be able to open this notebook for editing by running `jupyter notebook Introduction\ to\ phyx.js.ipynb` from the command line.

We use IJavascript to use Javascript as a kernel in Jupyter Notebook. If you would like to edit this notebook, you will need to install this as well.
